# Misdiagnosis of primary hepatic marginal zone B cell lymphoma of mucosa-associated lymphoid tissue type, a case report

**DOI:** 10.1186/s12957-016-0817-5

**Published:** 2016-03-08

**Authors:** L. X. Li, S. T. Zhou, X. Ji, H. Ren, Y. L. Sun, J. B. Zhang, H. B. Wang, Z. W. Liu

**Affiliations:** Center of Hepatobiliary Surgery and Liver Transplantation, 302 Hospital, 100 Xisihuang Middle Road, Fengtai District Beijing, 100039 China

**Keywords:** Extra-nodal marginal zone B cell lymphoma of mucosa-associated lymphoid tissue, CD20, Misdiagnosis, Left lateral sectionectomy

## Abstract

**Background:**

Extra-nodal marginal zone B cell lymphoma of mucosa-associated lymphoid tissue originating in the liver is less common.

**Case presentation:**

We described the clinical presentation, immunohistochemistry, and immunophenotypes of this lymphoma, which was misdiagnosed with tiny hepatic carcinoma in a 44-year-old woman with hepatitis C; the patient underwent left lateral sectionectomy. The immunophenotype identified the most of the lymphoid cells as positive CD20, CD34, Ki67, CD3, CD4, CD79a, CD45RO, MUM-1, and CD5 and negative CD10, CD15, CD30, ACT, CK, CRO, DES, and HMB45. The diagnosis of primary hepatic mucosa-associated lymphoid tissue (MALT) was made by histology after surgery; the patient went through the excellent recovery with no chemotherapy and is disease free for 27 months.

**Conclusions:**

Primary hepatic MALT is less common with incidental finding; local resection is beneficial due to its oncological indolence.

## Background

In 1983, Isaacson et al. first described four patients with primary low-grade hepatic B cell lymphoma of mucosa-associated lymphoid tissue as a distinct entity [1]; later, the lymphomas were sporadically reported and its prevalence is very low [2–4]. Jaffe documented that hepatic malignant lymphomas comprised less than 1 % of all malignant lymphomas, while hepatic mucosa-associated lymphoid tissue (MALT) lymphomas are reported to occur in only 3 % of cases of hepatic malignant lymphoma [5]. Due to the heterogeneous clinical presentations and no specific manifestations of MALT, this lymphoma was commonly misdiagnosed with liver cancer in the absence of biopsy [6, 7]. Herein, we first focused on a primary hepatic MALT-type lymphoma misdiagnosed as tiny liver cancer in a patient with hepatitis C virus (HCV)-associated cirrhosis who underwent left lateral sectionectomy.

## Case presentation

**Fig. 1:**
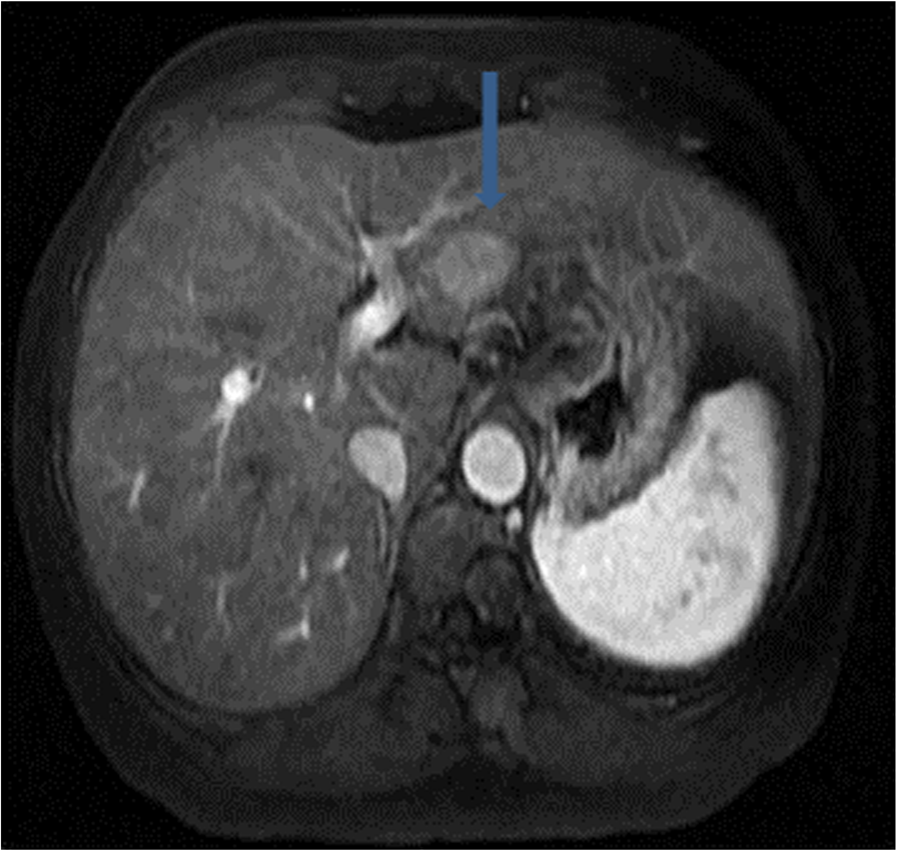
MRI of the liver in a patient with chronic liver disease. Arterial phase extracellular Gd-chelate-enhanced MRI demonstrating a T1 and T2 hyperintense sphenoid lesion of 1.8 cm (*arrow*) in diameter in the Sg 3 which is enhanced in the early phase

**Fig. 2:**
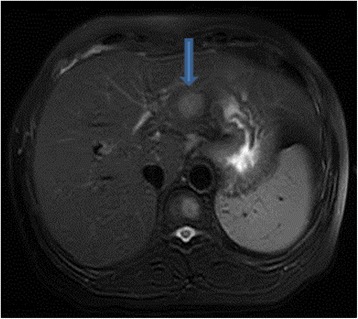
Portal phase extracellular Gd-chelate-enhanced MRI demonstrating a T1 and T2 hypointense sphenoid lesion (*arrow*) in the Sg 3 which is enhanced in the delayed phase, indicating a small hepatocellular carcinoma

**Fig. 3:**
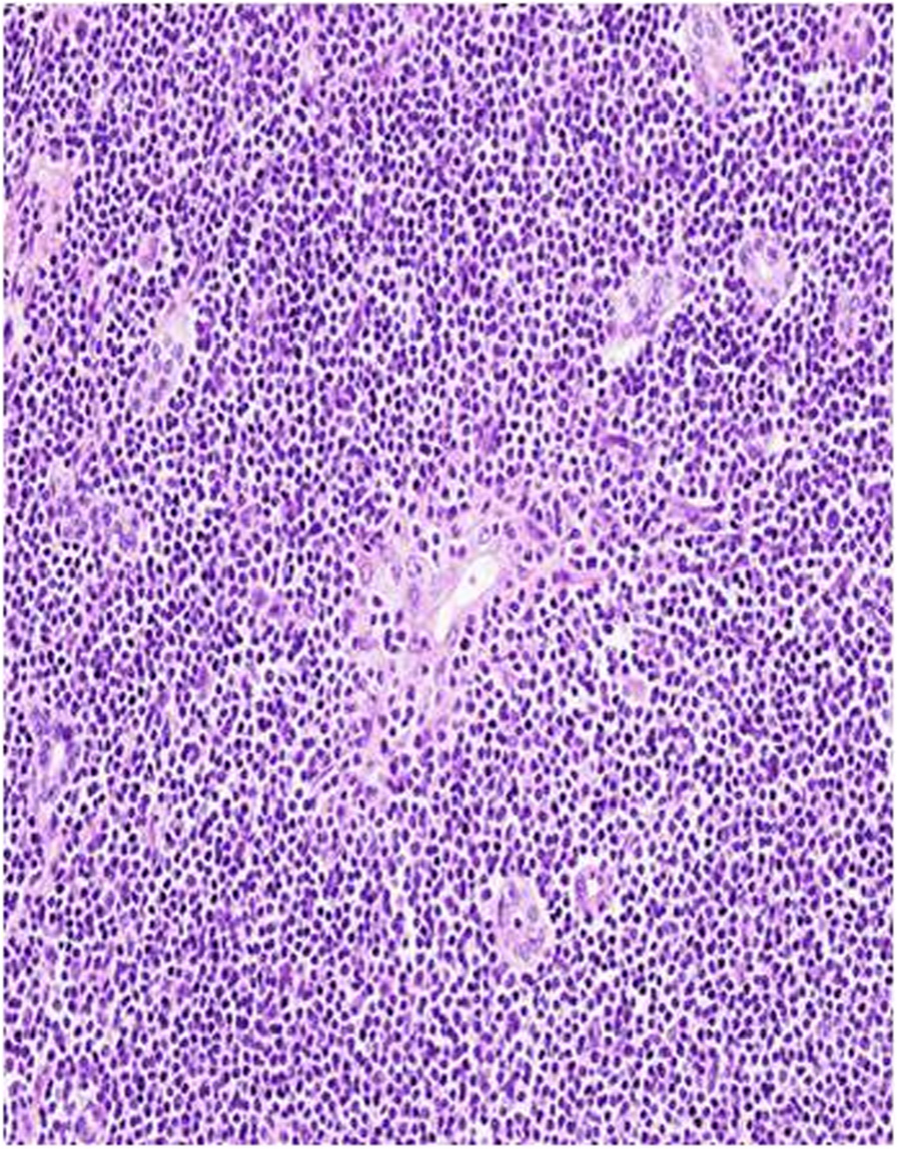
Lymphoid cells infiltrated in the cirrhosis liver

**Fig. 4:**
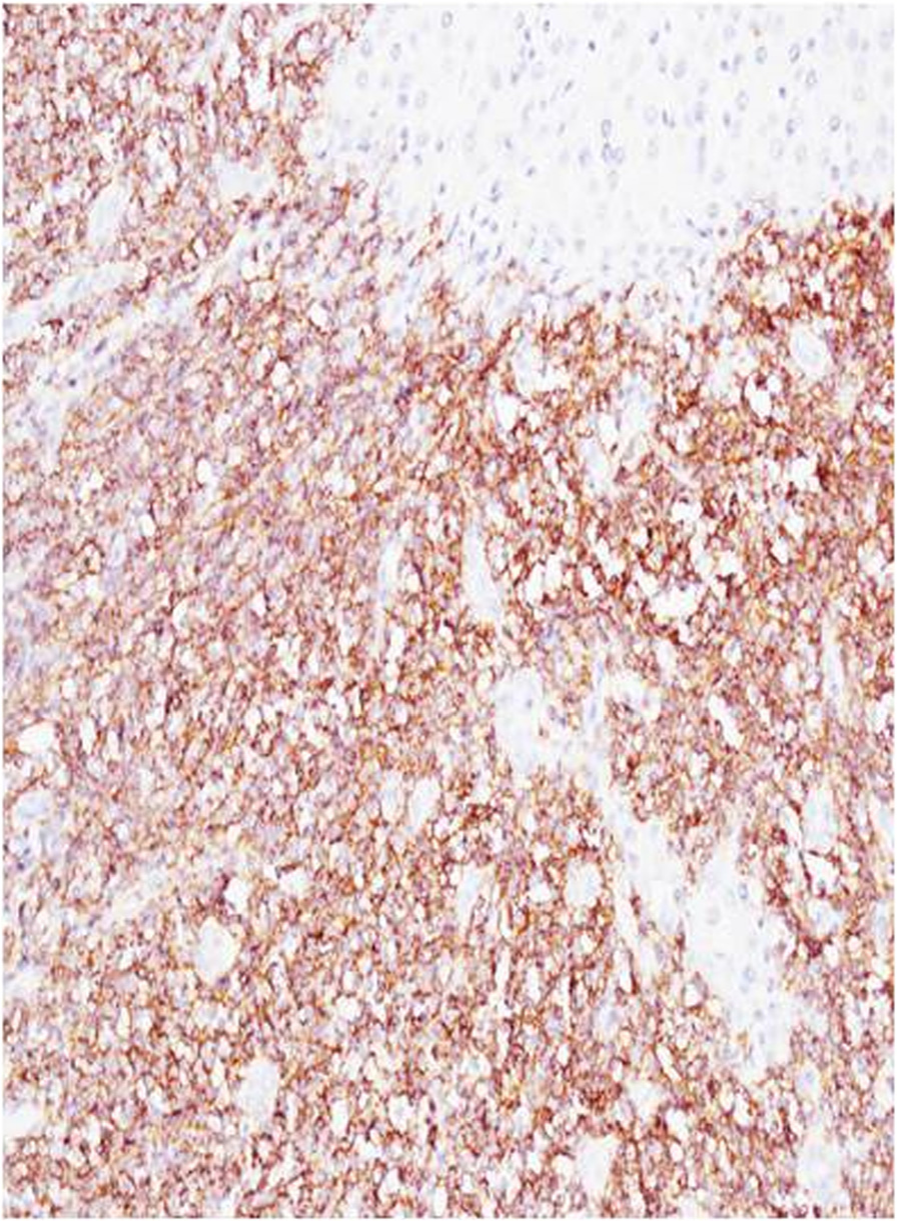
Most lymphoid cells positive for CD20 antibody

A 49-year-old female patient of Han Ethnicity presented with mild distention pain in the right hypochondrium; her disease history included hepatitis C for about 10 years, and at that time, liver functions test was performed, revealing aminotransferase lightly beyond the upper normal limit. On physical examination, laboratories were significant for positive HCV antibody, quantitative HCV RNA was 1.28 × 10^6^ cope/ml, gene subtype is 1b, and no regular treatment of HCV was recorded. Tumor biomarkers were unremarkable, including alpha fetoprotein, carbohydrate antigens, and carcinoembryonic antigen; autoimmune diseases test were negative, and thyroid immunoglobulin was within the normal range. Enhanced MRI demonstrated a T1 and T2 hyper-intensity round mass of 1.8 cm in the segment 3 of the liver which was enhanced in the early phase, clinically indicating tiny hepatocellular carcinoma (Figs. 1 and 2). Esphagogastroendoscopic findings indicated non-atrophic gastritis with erosions, and it was negative for *Helicobacter pylori* antibody. Polyp in the rectum was detected with colon endoscope, and biopsy revealed the inflammatory hyperplasia. The relevant cytogenetic testing was not performed, liver biopsy was not used to determine the imaging due to potential needle metastasis, and no periphery lymphadenopathy and splenomegaly were detected. Based on the history of chronic hepatitis C infection and almost typical imaging character, the diagnosis of tiny liver cancer was made, and left lateral sectionectomy was performed. Later pathology determined the primary hepatic marginal zone B cell lymphoma of mucosa-associated lymphoid tissue type. Its immunohistochemistry findings were positive for CD20(+++), CD5(+), CD34(+), Ki67(30 %+), CD3(+), CD4(++), CD79a(++), CD45RO(++), MUM-1(+++), and fewer Kappa(+) whereas they were negative for CD8(−), CD10(−), HBsAg(−), HBcAg(−), HCV(−), Hepa(−), GPC-3(−), CD15(−), CD30, CD56(−), lambda(−), and CB2(−) (Figs. 3 and 4). The patient experienced an uneventful recovery course. According to Ann Arbor staging, it was staged as IE. Chemotherapy was not applied even with the informed consent. The patient received combination of interferon β and ribavirin for 48 weeks, and HCV DNA test was negative. Later, positron emission tomography-CT revealed no relapse, and Hashimoto thyroiditis was confirmed upon the elevation of thyroid immunoglobulin. At follow-up, the woman was doing well for over 2 years.

### Discussion

The World Health Organization classified extra-nodal MALT lymphomas as low-grade ones characterized by their indolent, prolonged, localized, and clinical course and potential curability with local amendments [8]. The etiology of hepatic malignant lymphomas, especially MALT lymphomas, remains unknown. It was reported that *Helicobacter pylori*, hepatitis C virus, Epstein-Barr virus, and *Borrelia burgdorferi* were demonstrated in association with MALT lymphoma [9], and hepatitis C virus infection played a role in the occurrence of MALT reported by Ferri in 1993 [10]. Hashimoto’s thyroiditis represents a background for the development of 94 % of thyroid MALT lymphoma [11]. The molecular and cytogenetic data were very important, which not only contributed to a correct diagnosis but also shed light on the pathogenesis of this rare disease. The chromosomal translocations in MALT lymphoma was detected, and t(11;18)(q21;q21) is the most popular fusion signal in MALT lymphoma. B cell non-Hodgkin’s lymphoma (B-NHL) is a well-documented complication of HCV infection. For our case with HCV, the test for *H. pylorus* was negative, Hashimoto’s thyroiditis followed MALT, and molecular and cytogenetic tests were unavailable due to money budget in China. We think chronic inflammation by HCV hepatitis may involve many types of cells including lymphocytes, and insidiously oncogenesis occurs.

No specific immuno-histochemical marker has yet been identified for MALT lymphoma. The presence of CD20(+) is highly suggestive of lymphoma, and so is CD5(+) too. CD5(+) allow us to differentiate chronic lymphocytic leukemia from small lymphocytic lymphoma or mantle cell lymphoma. Cyclin D1-positivity closely fits the characteristics of classical, typical lymphocytic leukemia [12]. So, the evaluation of a panel of immunostains is necessary for the assessment of the architecture of the lymphoid infiltrate, lineage assignment, and identification of an aberrant phenotype and for the exclusion of other lymphomas and facilitating differential diagnosis [13]. This report suggested that clinicians should be concerned about the possibility of hepatic MALT in HCV patients with a hepatic tumor as a differential diagnosis.

Generally, the prevalence of hepatic malignant lymphomas is extremely low, and it lacks specific clinical presentations and biomarkers. The imaging feature was similar to that of hepatocellular carcinoma (HCC), so it is commonly misjudged as an HCC [14] as well as, in our case, misdiagnosed as tiny liver cancer, although there were no findings to indicate a malignant lymphoma in systemic screening and preemptive resection would confer greatly on the patients. It is time to distinguish HCC from hepatic malignant lymphoma by following their criteria [15]. Contrast-enhanced image studies including angiography, dynamic CT, or Sonazoid-enhanced ultrasound might bring effective findings to distinguish them. Most of the time, biopsy works as the gold standard for the diagnosis of liver tumors including MALT if necessary, though it may spread tumors. To date, it needs warranting intensive investigations and greater case accumulations.

Ann Arbor staging depends on both the place where the malignant tissue is located and the systemic symptoms due to the lymphoma. Staging and classification guide the treatment options, and the treatment of lymphoma (gastric) continues to evolve. Disappearance of MALT-type lymphoma in patients infected with HCV has been reported after anti-viral treatment with interferon and ribavirin [16]. Low-graded MALT is susceptible to resection while high-graded MALT carries poor prognosis due to early metastasis and low response to treatments. Our case was staged IE which indicated chemotherapy is not necessary, and no recurrence was detected for 27 months; long-lasting follow-up should be made to monitor if the patient is disease free.

A written informed consent was obtained from the patient for hepatectomy and potential publication of this case report and any accompanying images.

## Conclusions

Primary hepatic MALT is rare with incidental finding, and local treatment may be beneficial due to its oncological indolence.

## Abbreviations

HCV: hepatitis C virus
MALT: mucosa-associated lymphoid tissue
